# *ANO10* mutations cause ataxia and coenzyme Q_10_ deficiency

**DOI:** 10.1007/s00415-014-7476-7

**Published:** 2014-09-03

**Authors:** Andrea Balreira, Veronika Boczonadi, Emanuele Barca, Angela Pyle, Boglarka Bansagi, Marie Appleton, Claire Graham, Iain P. Hargreaves, Vedrana Milic Rasic, Hanns Lochmüller, Helen Griffin, Robert W. Taylor, Ali Naini, Patrick F. Chinnery, Michio Hirano, Catarina M. Quinzii, Rita Horvath

**Affiliations:** 1 Department of Neurology, Columbia University Medical Center, New York, NY USA; 2 Institute of Genetic Medicine, Newcastle University, Newcastle upon Tyne, UK; 3 Wellcome Trust Centre for Mitochondrial Research, Institute of Neuroscience, Newcastle University, Newcastle upon Tyne, UK; 4 Department of Molecular Neuroscience, UCL Institute of Neurology, London, UK; 5 Clinic for Neurology and Psychiatry for Children and Youth, Faculty of Medicine, University of Belgrade, Belgrade, Serbia; 6 Department of Pathology and Cell Biology, Columbia University Medical Center, New York, NY USA

**Keywords:** Autosomal recessive ataxia, Mitochondrial, Coenzyme Q_10_ (CoQ_10_) deficiency, *ANO10*

## Abstract

Inherited ataxias are heterogeneous disorders affecting both children and adults, with over 40 different causative genes, making molecular genetic diagnosis challenging. Although recent advances in next-generation sequencing have significantly improved mutation detection, few treatments exist for patients with inherited ataxia. In two patients with adult-onset cerebellar ataxia and coenzyme Q_10_ (CoQ_10_) deficiency in muscle, whole exome sequencing revealed mutations in *ANO10*, which encodes anoctamin 10, a member of a family of putative calcium-activated chloride channels, and the causative gene for autosomal recessive spinocerebellar ataxia-10 (SCAR10). Both patients presented with slowly progressive ataxia and dysarthria leading to severe disability in the sixth decade. Epilepsy and learning difficulties were also present in one patient, while retinal degeneration and cataract were present in the other. The detection of mutations in *ANO10* in our patients indicate that ANO10 defects cause secondary low CoQ_10_ and SCAR10 patients may benefit from CoQ_10_ supplementation.

**Electronic supplementary material:**

The online version of this article (doi:10.1007/s00415-014-7476-7) contains supplementary material, which is available to authorized users.

## Introduction

Despite major advances in understanding the genetic forms of ataxia, approximately half of the patients with recessive ataxia—in particular, adults—do not receive a molecular diagnosis [1]. Given the numerous etiologies, genetic diagnosis requires the performance of a wide range of analyses and consensus on the diagnostic value of these examinations is lacking. Recent advances in next-generation sequencing have significantly improved the mutation detection rate of inherited ataxias, but treatments for inherited ataxia remain inadequate. Clinical characterization and evaluation for subtle additional features can provide clues to the precise diagnosis [2].

Coenzyme Q_10_ (CoQ_10_) deficiencies comprise a heterogeneous group of autosomal recessive conditions with primary deficiencies caused by mutations in genes encoding CoQ_10_ biosynthesis enzymes and secondary forms caused by genetic defects not directly related to CoQ_10_ biosynthesis [3].Ataxia is the most common clinical phenotype associated with CoQ_10_ deficiency [3]. The first causative mutations identified in the ataxic form of CoQ_10_ deficiency were detected in *APTX,* which encodes aprataxin and is the causative gene for ataxia with oculomotor apraxia 1 (AOA1) [4]. Reduced CoQ_10_ levels were reported in some but not all AOA1 patients with confirmed pathogenic *APTX* mutations [4]. Autosomal recessive mutations in *ADCK3,* which encodes a kinase involved in CoQ_10_ biosynthesis, have been identified in several families with juvenile- or adult-onset ataxia, confirming the link between ataxia and CoQ_10_ deficiencies [3, 5]. Some patients improved on CoQ_10_ supplementation [3, 5]; the lack of improvement in some patients has been attributed to the reduced bioavailability of CoQ_10_ and its limited ability to cross the blood brain barrier; however, worsening has been reported for one patient treated with Idebenone, a short-chain CoQ analog [6].

In our genetic characterization of a group of patients with unexplained recessive or sporadic cerebellar ataxia and low muscle CoQ_10_, we identified pathogenic mutations in *ANO10* in two families.

## Case series

This study had institutional and ethical review board approval and all the patients gave informed consent.

## Patients and methods

We studied 40 unrelated patients with unexplained ataxia and low CoQ_10_ in skeletal muscle biopsies without a dominant family history, in whom well-known genetic causes of spinocerebellar ataxias (SCA1,2,3,6,7,17, DRPLA and Friedreich ataxia) and other possible causes of a progressive ataxia were excluded by clinical and laboratory investigations (brain MRI, CSF analyses, normal routine biochemistry, blood cell counts, metabolic screening for acyl-carnitine profiles, urine organic acids, very long chain fatty acids, phytanic acid, vitamins E, A, B12, alpha-fetoprotein, serum protein electrophoresis, lipoproteins, lysosomal enzymes, copper, ceruloplasmin, ferritin, iron, and paraneoplastic antibodies).

CoQ_10_ measurement in plasma, fibroblasts and skeletal muscle was performed as described [7].

### Molecular genetics

**Fig. 1:**
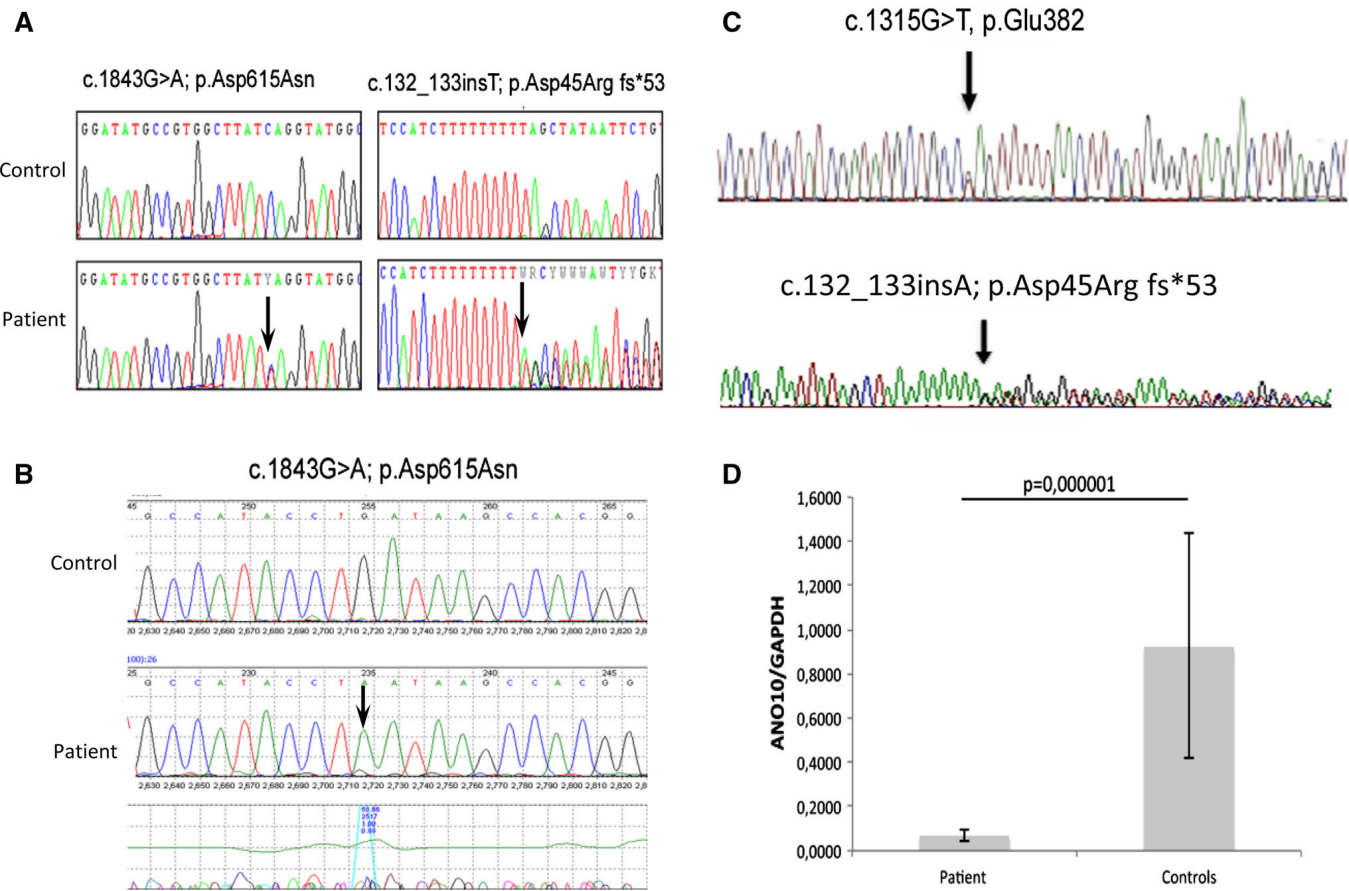
Detection of heterozygous *ANO10* variants in genomic DNA of patient 1 (**a**). cDNA analysis of patient 1 detected the c.1843G>A, p.Asp615Asn mutation in hemizygous form, confirming compound heterozygosity (**b**), Compound heterozygous mutations were detected in patient 2 (**c**), Q-RT-PCR showed significantly decreased ANO10 mRNA levels in patient 2’s fibroblast, compared with controls (**d**). Values are expressed as mean ± SD of patient and controls (*N* = 3)

Genetic analysis of mitochondrial DNA (mtDNA) for deletions, depletion, and point mutations in muscle DNA as well as direct sequencing of *POLG,* *PDSS1, PDSS2, COQ2, COQ9, CABC1/ADCK3* and *APTX* in blood DNA were normal in all patients [3]. In patient 1, whole exome sequencing was performed in genomic DNA, isolated from lymphocytes (DNeasy^®^, Qiagen, Valencia, CA), fragmented and enriched by Illumina TruSeq™ 62 Mb exome capture, and sequenced (Illumina HiSeq 2000, 100 bp paired-end reads). The in-house bioinformatics pipeline included alignment to the human reference genome (UCSC hg19), reformatting, and variant detection (Varscan v2.2, Dindel v1.01), as described previously [8]. On-target variant filtering excluded those with minor allele frequency greater >0.01 in several databases. Rare homozygous and compound heterozygous variants were defined, and protein altering and/or putative ‘disease causing’ mutations, along with their functional annotation, were identified using ANNOVAR [8]. Putative pathogenic variants were confirmed by Sanger sequencing, using custom-designed primers (http://frodo.wi.mit.edu) on an ABI 3130XL (Life Technologies, CA, USA), allowing segregation analyses (Fig. 1a). The primers used for genomic DNA (NG_028216.1) and cDNA (NM_018075.3) analysis of *ANO10* are listed in the Supplementary Materials (Supplemental Tables 1 and 2).

To measure *ANO10* mRNA expression level, skin fibroblasts from the patients were grown and total RNA was extracted using the Pure Link™ RNA Mini Kit (Ambion, Life Technologies). RNA concentration was measured using a Nanodrop. Subsequently, 100 ng of RNA was converted into cDNA using SuperScript VILO cDNA Synthesis Kit (Invitrogen). Quantitative RT-PCR (qRT-PCR) was performed using TaqMan Assays for *ANO10* and *GAPDH* (Applied Biosystems).

## Results

Patient 1 is a 57-year-old English woman who is the second child of healthy, non-consanguineous parents and has a healthy brother. After normal early development she developed generalized epilepsy and learning difficulties at 7 years of age. Her epilepsy was well-controlled, but at age 45 years, she developed gait disturbance, ataxia and slurred speech. She became wheelchair-bound from age 50 years. On examination she had slow saccades but no nystagmus, dysarthria, bilateral dysmetria and a severe trunk ataxia. Although she had no weakness and deep tendon reflexes were normal, she was permanently wheelchair-bound and could not stand or walk due to severe trunk ataxia and had some cognitive dysfunction. Brain MRI revealed marked parieto-occipital and cerebellar atrophy. Nerve conduction studies and electromyography were normal. Skeletal muscle biopsy was performed at age 53 years and showed normal histology, but an isolated complex III deficiency (158 normalize to citrate synthase, normal mean ± standard deviation 554 ± 345) and low CoQ_10_ (104 pmol/mg protein, normal range 140–580). CoQ_10_ in fibroblasts was normal (147 pmol/mg protein, normal range 75–190 pmol/mg protein).

Supplementation with 1,000 mg/day CoQ_10_ had beneficial effects on the patient’s fatigue and mobility within 3–4 months. She was able to stand up and do a few steps and her cognition has slightly improved. She had no seizures and no epileptic activity on her follow-up EEG, therefore, antiepileptic medication was slowly withdrawn.

### Genetic analysis

Whole exome sequencing detected potentially disease causing variants in nine genes, but only the variants in *ANO10* segregated with the disease and alter a known disease gene. Two potentially disease causing variants (c.132_133insT; p.Asp45Arg fs*53 and c.1843G>A; p.Asp615Asn) were detected in *ANO10* (Fig. 1a), which were not present in her healthy brother. Unfortunately, both parents of patient 1 had died and she did not have any children. One of the detected mutations was a frame-shift mutation leading to a premature stop codon. The other mutation c.1843G>A; p.Asp615Asn is a missense change of a conserved amino acid that has been detected extremely rarely (rs138000380—1,000 genomes shows only 1 heterozygous) and both mutation taster (0.999) and PolyPhen (0.559) predicted that it is disease causing. cDNA analysis for *ANO10* detected only one allele, which was supported by the hemizygous state of the c.1843G>A; p.Asp615Asn change (Fig. 1b).

The detection of pathogenic *ANO10* mutations in our patient with the ataxic form of CoQ_10_ deficiency prompted us to perform sequencing of *ANO10* in 36 further patients with low CoQ_10_ in muscle, fibroblasts, or CSF [3], and we detected pathogenic mutations in one additional patient.

Patient 2 is a 52-year-old woman with neurologically normal non-consanguineous parents, and two healthy siblings. At the age of 30 years, she presented with walking difficulties, slurred speech, and oscilloscopia. The walking instability progressed slowly and she developed retinal detachments that were treated with multiple surgeries. At age 50, because of frequent falls, she started to use a walker to ambulate long distance. Other ocular problems include: retinal fibrosis, cataracts, vitreous fluid opacity and possible macular degeneration. Neurological examination revealed a stiff-legged wide-based gait with scissoring, slight eyelid ptosis, primary gaze down-beat nystagmus, and brisk tendon reflexes with ankle clonus, crossed hip adductors, and bilateral Hoffmann signs but Babinski signs were absent. Dysmetria and intention tremor on finger-nose-finger and heel-to-shin test were present. Brain MRI revealed marked cerebellar atrophy involving both lobes and the vermis. Muscle biopsy showed fiber type 2 atrophy. Respiratory chain complex activities measured in muscle tissue were normal.

CoQ_10_ level in the fibroblasts of the patient (50.6 ± 7.3 nmol/μg protein) was normal (concurrent controls 48.3 ± 27.8 nmol/μg protein); however, levels of CoQ_10_ were decreased in plasma (0.41 μg/ml, normal range 0.8 ± 0.35 μg/ml) and CSF (410 μg/l, normal range 450–1420 μg/l); and borderline in muscle (21.2 μg/g, normal range 32 ± 6 μg/g).

CoQ_10_ supplementation (120–180 mg/day) led to an initial mild improvement of the ataxia and gait. Evaluation by the international co-operative ataxia rating scale (ICAR), showed an improvement in the oculomotor movement score (from 4/6 to 3/6) and in the dysarthria score (from 6/8 to 5/8).

### Genetic analysis

Direct sequencing of *ANO10* in genomic DNA from patient 2 revealed two heterozygous variants, c.132_133insT; p.Asp45Argfs*53 in exon 2, also present in patient 1, and c.1315G>T, p.Glu382*, in exon 6 (Fig. 1d). Both variants generate premature stop codons with truncated protein of 53 aa and 382 aa, respectively (wild-type protein: 660 aa).

The 2 mutations segregated with the disease in the family (the mother was heterozygous for the c.132_133insT; p.Asp45Argfs*53 variant, both healthy siblings were homozygous wild type; and the father’s DNA was not available) and were not detected in 124 ethnically-matched control alleles. cDNA analysis showed a significant reduction of *ANO10* expression level in cultured skin fibroblast from patient 2 compared to controls (Fig. 1d).

Measurement of CoQ10 in fibroblasts of a previously published patient (family B, II:5) [9] in carrying pathogenic *ANO10* mutations was normal result (156 pmol/mg protein, normal range 75–190 pmol/mg protein), however, muscle was not available for CoQ_10_ analysis.

## Discussion

Anoctamin 10 (ANO10), also known as TMEM16 K, is a member of the human anoctamin (ANO) family of proteins, which consists of at least nine other proteins, all containing eight transmembrane domains and a DUF590 domain [9, 10]. It has been suggested that the anoctamin genes encode cell- and tissue-specific calcium-activated chloride channels, however, experimental data are limited [10]. The clinical presentations of mutations in the various anoctamin genes are very heterogeneous, and include limb-girdle muscular dystrophy (ANO5), skeletal abnormalities (Gnathodiaphyseal dysplasia due to *ANO5* mutations), blood cell disorders (Scott syndrome caused by *ANO6* defects), as well as progressive neurological presentations of autosomal dominant dystonia (DYT24 due to *ANO3* mutations) and cerebellar ataxia and atrophy (*ANO10* defects) [10].

**Fig. 2:**
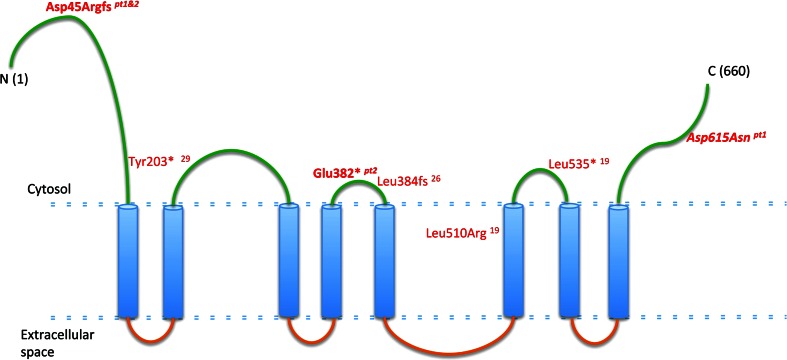
Schematic representation of ANO10. In *green*: putative intra-cytoplasmic region, in *blue*: putative transmembrane domains, in *orange*: putative extracellular domains. Text in *red*: previously reported mutations; in *bold italic*: mutations found in our patients

Mutations in *ANO10* (Fig. 2) has been associated with autosomal recessive cerebellar ataxia in only five families [9, 11, 12]. The clinical presentation of the previously reported patients showed cerebellar ataxia and atrophy with variable age-at-onset between 13 and 45 years, brisk reflexes, and eye movement abnormalities. Additional features included: intellectual deficit, motor neuron involvement and epilepsy in some but not all patients, illustrating the significant clinical variability (Table 1). Although the clinical presentation of our patients resembles the previously reported cases, and confirms the clinical phenotype of ANO10 deficiency, muscle biopsies and measurement of CoQ_10_ have never been assessed before in this condition. Because 2 out of 40 patients (5 %) from our cohort carried mutations in *ANO10*, we suggest that defects in this gene should be considered in patients with unexplained ataxia and low CoQ_10_ in skeletal muscle.

**Table 1:** Clinical summary of patients 1 and 2 reported in this study and the previously reported patients carrying pathogenic *ANO10* mutations

Ref	Age of onset (years)	Country of origin	Genotype	Gait ataxia	Dysarthria	Limb ataxia	Cortico-spinal tract	MRI	Opthalmologic features	EMG	Mental retardation	Other features
6	25	Netherlands	c.1529T>G; p.Leu510Arg	++	++	++	↑DTR EPR	CA	Downbeat nys	II MU involvement	–	Cold and blue toes
6	20	Netherlands	c.1529T>G; p.Leu510Arg	++	++	++	↑DTR	CA	Downbeat nys	II MU involvement	–	Wasting and fasciculations proximal leg muscles
6	32	Netherlands	c.1529T>G; p.Leu510Arg	+	+	+	↑DTR	CA	Downbeat nys	NP	–	Cold and blue toes
6	15	Serbia	c.1150_1151del; p.Leu384 fs	++	++	+	↑DTR	CA	Hor and ver nys	NP	+	Inspiratory stridor
6	15	Serbia	c.1150_1151del; p.Leu384 fs	++	+	+	↑DTR	CA	Hor and ver nys	NP	+	Pes cavus
6	13	Serbia	c.1150_1151del; p.Leu384 fs	++	++	+	↑DTR	CA	Hor nys	II MU involvement	–	Fasciculations proximal leg muscles, inspiratory stridor, vocal cord paresis
6	45	France	c.1476 + 1G>T c.1640del; p.Leu535*	+++	++	++	↑DTR	NP	Saccadic pursuit, nys	NP	–	Rest tremor, pes cavus
6	25	France	c.1476 + 1G>T c.1640del; p.Leu535*	++	++	++	↑DTR	CA	Multi-dir nys	Normal	–	Episodic diplopia, pes cavus
8	16	Bulgaria	c.1150_1151del; p.Leu384 fs	+++	++	+	↑DTR	NP	Hor and ver nys	NP	–	NR
8	17	Bulgaria	c.1150_1151del; p.Leu384 fs	++	++	+	↑DTR EPR	CA	Downbeat nys	II MU involvement	+	NR
8	17	Bulgaria	c.1150_1151del; p.Leu384 fs	++	++	+	↑DTR	NP	Downbeat nys	Normal	+	NR
9	46	Japan	c.609C>G; p.Tyr203*	++	++	NA	↑DTR	Mild CA	Saccadic pursuit	Normal	–	Seizures, constipation
P1 (this paper)	7	United Kingdom	c.132_133insT; p.Asp45Argfs c.1843G>A; p.Asp615Asn	+++	++	++	Normal	Parieto-occipital, CA	Saccadic pursuit	Normal	+	Seizures, Low CoQ10 in muscle
P2 (this paper)	30	United States	c.132_133insT; p.Asp45Argfs c.1315G>T; p.Glu382*	++	++	++	↑DTR Hoffman sign	Marked CA	Downbeat nys	Normal	–	Retinal degen, cataract, low CoQ10 in blood and CSF

*Ref* reference, Age of onset (years), *MRI* magnetic resonance imaging, + Mild, ++ Moderate,+++ Severe, ↑ increased, *DTR* deep tendon reflexes, *EPR* extension plantar reflex, *NP* not preformed, *hor* horizontal, *ver* vertical, *nys* nystagmus, *Multi-dir* multi-directional, *MU* motor unit, *NA* not available, *CoQ10* coenzyme Q10, *CSF* cerebral spinal fluid

The low CoQ_10_ levels and the beneficial effect of CoQ_10_ supplementation in our patients carrying *ANO10* mutations are unexpected findings and raise the question whether low CoQ_10_ contributes to the disease pathomechanism, potentially by affecting CoQ_10_ dependent functions, which was supported by slightly low CoQ_10_ in fibroblasts of a previously reported patient [9]. Interestingly, in one of our patients, seizures disappeared after CoQ_10_ supplementation, as previously reported in a patient with CoQ_10_ deficiency and cerebellar ataxia due to *APTX* mutations [4]. It has been postulated that cerebellar ataxia in patients with ANO10 deficiency may be due to abnormal calcium signaling in Purkinje cells [10]. Calcium signaling has been shown to be important for mitochondria, and this year, mutations in the gene encoding for the mitochondrial calcium uptake 1 (MICU1) protein have been identified in some families with proximal myopathy, learning difficulties and a progressive extrapyramidal movement disorder [13]. The role of ANO10 and CoQ_10_ in calcium signaling need to be further investigated. Functional studies in additional patients or animal models will be helpful to characterize the pathomechanism of *ANO10* mutations and will define the role of CoQ_10_ deficiency in ANO10-related disease. The observations of low CoQ_10_ levels and the beneficial effect of high-dose CoQ_10_ supplementation in our patients suggest that CoQ_10_ should be considered in the therapy of ANO10 deficiency.

## Electronic supplementary material

Below is the link to the electronic supplementary material.

Supplementary material 1 (DOCX 22 kb)
