# Supplementary material for: ANO10 mutations cause ataxia and coenzyme Q10 deficiency
Source: J Neurol. 2014 Sep 3;261(11):2192–8. doi: 10.1007/s00415-014-7476-7 (PMC4221650; doi:10.1007/s00415-014-7476-7)
Supplement: Supplementary file 1 — Supplementary material 1 (DOCX 22 kb) [file 415_2014_7476_MOESM1_ESM.docx]

**Journal of Neurology**

**Supplementary Material**

***ANO10* mutations cause ataxia and coenzyme Q_10_ deficiency**

Andrea Balreira^1^, Veronika Boczonadi*^2*^*, Emanuele Barca^1^, Angela Pyle*^2^*, Boglarka Bansagi*^2^*, Marie Appleton^3^, Claire Graham*^2^*, Iain P. Hargreaves^4^, Vedrana Milic Rasic^5^, Hanns Lochmüller^2^, Helen Griffin*^2^*, Robert W. Taylor*^3^*, Ali Naini^6^, Patrick F. Chinnery*^2,3^*, Michio Hirano^1^, Catarina M Quinzii^1#^, Rita Horvath*^2#^*

# *^1^Department of Neurology, Columbia University Medical Center, New York, NY, USA;*

*^2^Institute of Genetic Medicine, Newcastle University, Newcastle upon Tyne, UK;*

*^3^Wellcome Trust Centre for Mitochondrial Research; Institute of Neuroscience, Newcastle University, Newcastle upon Tyne, UK;*

*^4^Department of Molecular Neuroscience, UCL Institute of Neurology, London, UK;*

*^5^Clinic for Neurology and Psychiatry for Children and Youth, Faculty of Medicine, University of Belgrade, Serbia;*

***^6^****Department of Pathology and Cell Biology, Columbia University Medical Center, New York, NY, USA*

**Corresponding authors**:

Rita Horvath, MD PhD

Phone: +44 191 2418855, Fax: +44 191 2418666, Email: [Rita.Horvath@ncl.ac.uk](mailto:Rita.Horvath@ncl.ac.uk)

Catarina M Quinzii, MD

Phone: +12123421296, Fax: +12123053986, Email: [cmq2101@cumc.columbia.edu](mailto:cmq2101@cumc.columbia.edu)

**Supplemental Table 1: Genomic DNA sequencing of *ANO10***

PCR program: 5 min at 94ºC; 35 cycles of 30s at 94ºC, 30 s at Tm annealing, 1 min at 72ºC; 7 min at 72ºC. The primers sequences for genomic DNA sequencing were based on the ones reported in Vermeer S *et al,* 2010, with few exceptions.

| FRAGMENT (FRAGMENT SIZE) |  | SEQUENCE | ANNEALING TEMPERATURE (ºC) |
| --- | --- | --- | --- |
| **2** (327 bp) | F | TGCTTTTATCTTGGAAGCCAG | 56 |
|  | R | GGGAGGCTGAGCATACAGTG |  |
| **3** (457 bp) | F | AAAGAACTGCCATCCCTAATG | 60 |
|  | R | AAAAAGTTTGCTGATCCCTGA |  |
| **4** (744 bp) | F | CATACTGCTTTCTGCTCATTGG | 55 |
|  | R | ATTTTCATGTACAATGTTAGGGC |  |
| **5** (275 bp) | F | TGAAGCGTATCATGCACAATC | 59 |
|  | R | ATATCTGCCCAAGGGAGCTG |  |
| **6A** (459 bp) | F | AGGGTTGAATGATCCCCAC | 55 |
|  | R | AAATGCGCAACTGTCTCTTG |  |
| **6B** (435 bp) | F | AACATGACCTACAGGTGGGG | 55 |
|  | R | TGTGAATCCCATGATCTAGGC |  |
| **7** (304 bp) | F | GACTGAGGCTCTGATGTTGG | 56 |
|  | R | TCAATCCTTGCCTATTTGCAC |  |
| **8** (399 bp) | F | AGGCTGGGAGCTGTAGACTG | 59 |
|  | R | GCATAATACACAATGCCATTCC |  |
| **9** (569 bp) | F | GAAACACCATTCTAACACCTAGC | 56 |
|  | R | AGCAGTGCTTCAATGCAAAG |  |
| **10** (341 bp) | F | AGAGGCCACAGCTTTGATTAG | 55 |
|  | R | TTTCCCTGTCATAACACCTCG |  |
| **11** (290 bp) | F | AGGATGAGGAAATATGGAAGC | 55 |
|  | R | TTGCTCAATTGTCAGTCATGG |  |
| **12** (404 bp) | F | GGCCTGCTTGGTCTTTGATAC | 56 |
|  | R | TCCTGAACTGGAGTCCTCTG |  |
| **13A** (490 bp) | F | GCTTCCACAGAGAGCAGAGG | 56 |
|  | R | ACTGCTATGAGGGGAACGTG |  |
| **13B** (499 bp) | F | CCTTCTCAGTTTCGCAGTGG | 56 |
|  | R | ATCTCACCGCTCCACCTTC |  |

**Supplemental Table 2: Primers used for cDNA sequencing**

| FRAGMENT (FRAGMENT SIZE) |  | SEQUENCE | ANNEALING TEMPERATURE (ºC) |
| --- | --- | --- | --- |
| **1** (187 bp) | F | TTTCACACCTTTGGTGGTCA | 57 |
|  | R | CTTGGAGGCACCAACAAGAT |  |
| **2** (431 bp) | F | GAGGTGCCCAGTTGTTGTTT | 57 |
|  | R | CCGAGTGTACCAGGTGTCCT |  |
| **3** (599 bp) | F | GGCATCGTGATTCAGGTGTT | 57 |
|  | R | ATGCTGGGCACATACAACAG |  |
| **4** (580 bp) | F | GGGTCTGAGTGGACCAGTGT | 57 |
|  | R | GACGTTTGAAGACCCTGCAC |  |
| **5** (446 bp) | F | CAGCTGCCTTTGCTGTGTTA | 57 |
|  | R | TCCTTCAGGTTCTCGGTCAC |  |
|  | R | ATCTCACCGCTCCACCTTC |  |
